# TANK shapes an immunosuppressive microenvironment and predicts prognosis and therapeutic response in glioma

**DOI:** 10.3389/fimmu.2023.1138203

**Published:** 2023-05-05

**Authors:** Shasha Li, Youwei Guo, Huijuan Hu, Na Gao, Xuejun Yan, Quanwei Zhou, Hui Liu

**Affiliations:** ^1^ National Engineering Research Center of Ophthalmology and Optometry, Eye Hospital, Wenzhou Medical University, Wenzhou, China; ^2^ Oujiang Laboratory (Zhejiang Lab for Regenerative Medicine, Vision and Brain Health), Wenzhou, China; ^3^ Department of Neurosurgery, Xiangya Hospital, Central South University, Changsha, China; ^4^ State Key Laboratory of Ophthalmology, Optometry and Visual Science, Eye Hospital, Wenzhou Medical University, Wenzhou, China; ^5^ Department of Geriatrics, National Key Clinical Specialty, Guangzhou First People’s Hospital, Guangzhou Medical University, Guangzhou, China; ^6^ The National Key Clinical Specialty, Department of Neurosurgery, Zhujiang Hospital, Southern Medical University, Guangzhou, China

**Keywords:** glioma, immunosuppressive microenvironment, immune infiltration, TANK, prognosis

## Abstract

**Background:**

Glioma, the most prevalent malignant intracranial tumor, poses a significant threat to patients due to its high morbidity and mortality rates, but its prognostic indicators remain inaccurate. Although TRAF-associated NF-kB activator (TANK) interacts and cross-regulates with cytokines and microenvironmental immune cells, it is unclear whether TANK plays a role in the immunologically heterogeneous gliomas.

**Methods:**

TANK mRNA expression patterns in public databases were analyzed, and qPCR and IHC were performed in an in-house cohort to confirm the clinical significance of TANK. Then, we systematically evaluated the relationship between TANK expression and immune characteristics in the glioma microenvironment. Additionally, we evaluated the ability of TANK to predict treatment response in glioma. TANK-associated risk scores were developed by LASSO-Cox regression and machine learning, and their prognostic ability was tested.

**Results:**

TANK was specifically overexpressed in glioma and enriched in the malignant phenotype, and its overexpression was related to poor prognosis. The presence of a tumor microenvironment that is immunosuppressive was evident by the negative correlations between TANK expression and immunomodulators, steps in the cancer immunity cycle, and immune checkpoints. Notably, treatment for cancer may be more effective when immunotherapy is combined with anti-TANK therapy. Prognosis could be accurately predicted by the TANK-related risk score.

**Conclusions:**

High expression of TANK is associated with the malignant phenotype of glioma, as it shapes an immunosuppressive tumor microenvironment. Additionally, TANK can be used as a predictive biomarker for responses to various treatments and prognosis.

## Introduction

Glioma is the most common malignant intracranial tumor, and more than 60% of primary central nervous system (CNS) tumors (1) with a diffusely invasive nature (2). The median overall survival time for patients with gliomas ranges from ~14 months (3) to less than 10 years (4), giving rise to severe morbidity and mortality of patients. Over the past decade, remarkable advances in molecular profiling studies (5, 6) have deepened the understanding of the classification, diagnosis, treatment, and prognosis of glioma. As such, the 2016 WHO classification of glioma incorporated morphological and molecular features for division of gliomas into distinct subgroups (7) for precision diagnosis and treatment. In 2021, the latest version of the WHO classification subdivided diffuse glioma into adult-type and pediatric-type (8), with adult-type comprising three subtypes characterized by histological features and genetic mutation status. The current standard therapeutic strategies are surgical resection, radiotherapy, and chemotherapy, whereas combined therapy demonstrates modest efficacy. Physiological barriers, chemo- and radioresistance, and the paucity of clear targeted pathways contribute to the limitations of these treatments (9, 10). Consequently, novel treatment modalities focused on improving the life expectancy of glioma patients are urgently needed.

Accompanied by a deeper understanding of glioma biology, numerous preclinical and clinical trials have explored immunotherapies, such as immune checkpoint inhibitors (ICIs) (11), oncolytic viral therapies (12), adoptive cellular therapies (13), cytokine therapies (14) and vaccinations (15). The immune checkpoint pathway, integral to modulating self-tolerance and immune responses, serves as a major mechanism by which glioma escapes immunosurveillance and maintains immune resistance (16). ICIs restore tumoricidal activities by targeting coinhibitory molecules such as CTLA-4 and PD-1and have demonstrated encouraging efficacy in clinical trials of metastatic melanoma (17), Hodgkin’s lymphoma (18), HCC (19), and NSCLC (20). Various preclinical trials of ICIs or ICIs combined with other strategies in patients with glioma have been explored and found to have potential clinical value (21–24). Considering the paucity of strong clinical evidence and the exact mechanisms of action, the current state of knowledge emphasizes the urgency of exploring primary therapeutic approaches in combination with novel therapeutic targets to prolong the survival of glioma patients.

TRAF-associated NF-kB activator (TANK) is a protein with dual functions in activating NF-kB (25–27) that is indispensable for immune responses and inflammatory processes, as well as for activating survival and proinflammatory genes within the tumor microenvironment (28–30). Subsequent studies identified TANK as an adaptor protein that interacts with canonical IKKs (NEMO and IKKγ) (31) and IKK-related kinases (TBK1 and IKKϵ) (32) to modulate NF-κB and TLR-induced antiviral pathways and prevent autoimmunity (32, 33). Consequently, the immunological functions that TANK perform in the pathophysiological process of these diseases, especially cancers, deserve in-depth discussion.

Downregulated genes, including TANK/I-TRAF, were analyzed in HPV-16 E6-transfected carcinoma cells (34), whereas treatment with the antiproliferative agent cisplatin reversed the condition and downregulated the TRAF2-mediated NF-κB activity (35), indicating the tumorigenic properties of TANK and novel therapeutic targets for cervical cancer. Deregulated expression of TANK not only orchestrates the signaling network of the ERK1/2, AKT and IRF3 pathways in controlling the survival, proliferation, migration and invasion of glioblastoma (GBM) cells but also mediates the relative expression of genes in inflammatory signaling cascades (36). Moreover, TANK indirectly phosphorylates the transcription factor STAT3 to increase the release of interleukin-6 (IL-6) and ultimately accelerate the progression of glioma in terms of enhanced angiogenesis and proliferation. TANK interacts with and cross-regulates cytokines and microenvironmental immune cells, but its exact biological function remains uncertain and needs to be further investigated.

## Materials and methods

### Obtaining and processing data

The methods used for obtaining and processing data are the same as those described in previous literature (37). All data were downloaded from Chinese Glioma Genome Atlas (CGGA) datasets (CGGA-693, CGGA-325, CGGA-301), TCGA, GSE16011 and Rembrandt datasets.

### Human specimens

We retrospectively defined two cohorts from Xiangya Hospital, Central South University. Cohort 1 included 29 normal tissues and 200 glioma tissues for examining the mRNA expression of TANK by qPCR. Cohort 2 included 23 normal tissues and 203 glioma tissues for examining the protein expression of TANK by IHC. The relevant information can be seen in **Table 1**. Informed consent was obtained from all patients. Ethical approval was obtained for this study.

**Table 1:** Clinical characteristics of Cohort 1 and Cohort 2.

		Cohort 1 (n=200)	Cohort 2 (n=203)
Age (Mean ± SD)		43.98 ± 15.78	45.02 ± 15.90
Gender, n (%)	Female	83 (41.5%)	87 (42.9%)
	Male	112 (56%)	116 (57.1%)
	Unknown	5 (2.5%)	0 (0%)
WHO grade, n (%)	2	57 (28.5%)	52 (25.6%)
	3	36 (18%)	42 (20.7%)
	4	84 (42%)	95 (46.8%)
	Unknown	23 (11.5%)	14 (6.9%)
IDH1 status, n (%)	Mutant	67 (33.5%)	71 (35%)
	Unknown	22 (11%)	23 (11.3%)
	Wild-type	111 (55.5%)	109 (53.7%)
Histology, n (%)	Astrocytoma	74 (37%)	85 (41.9%)
	Gangliocytoma	5 (2.5%)	5 (2.5%)
	GBM	82 (41%)	96 (47.3%)
	Oligodendroglioma	24 (12%)	17 (8.4%)
	Unknown	15 (7.5%)	0 (0%)
Radiotherapy, n (%)	No	50 (25%)	55 (27.1%)
	Unknown	54 (27%)	30 (14.8%)
	Yes	96 (48%)	118 (58.1%)
Chemotherapy, n (%)	No	44 (22%)	55 (27.1%)
	Unknown	54 (27%)	30 (14.8%)
	Yes	102 (51%)	118 (58.1%)
Laterality, n (%)	Both	5 (2.5%)	6 (3%)
	Left	86 (43%)	89 (43.8%)
	Middle	10 (5%)	7 (3.4%)
	Right	96 (48%)	101 (49.8%)
	Unknown	3 (1.5%)	0 (0%)
Tumor location, n (%)	Brainstem	3 (1.5%)	3 (1.5%)
	Cerebellar	10 (5%)	7 (3.4%)
	Frontal	87 (43.5%)	93 (45.8%)
	Insular	6 (3%)	5 (2.5%)
	Occipital	11 (5.5%)	14 (6.9%)
	Parietal	18 (9%)	22 (10.8%)
	Sellar	3 (1.5%)	2 (1%)
	Temporal	55 (27.5%)	54 (26.6%)
	Thalamus	4 (2%)	3 (1.5%)
	Unknown	3 (1.5%)	0 (0%)

### RNA extraction and quantitative real-time PCR

We carried out these processes by referring to the previous study (37). The relative expression of TANK was calculated to ACTB by 2^–ΔΔCt^ method. Primer sequences are given below:

TANK (F) 5′- CCACTTCTGGACCCATCTGATG-3′,

TANK (R) 5′- GCAGTTCTGAGTCTGTGCCACT-3′,

ACTB (F) 5′-ACAGAGCCTCGCCTTTGCCGAT-3′,

ACTB (R) 5′- CTTGCACATGCCGGAGCCGTT-3′.

### Immunohistochemistry (IHC)

A TMA was constructed from 23 normal tissues and 203 glioma tissues. With reference to the previous literature (37), we conducted experiments. IHC assay was performed with primary antibodies against PD-1 (Proteintech, China), HIF1A (CST, United States), CD11b (AiFang, China), CD40 (Proteintech, China), PD-L1 (CST, United States), CD163 (Proteintech, China), STAT3 (Proteintech, China), and TANK (Bioss, China).

### Immunological characteristics of the glioma microenvironment

The immunological characteristics of the glioma microenvironment were evaluated by considering the expression levels of immunomodulators and infiltration levels of TIICs, the activity of the tumor immunity cycle, and inhibitory immune checkpoints. Date for a total of 122 immunomodulators, such as MHC, chemokines and immune stimulators, were obtained from previous studies (38). By single sample gene set enrichment analysis (ssGSEA), the seven steps of the tumor immunity cycle were assessed based on the gene expression profiles (39). Seven algorithms, including MCP-counter, CIBERSORT, quanTIseq, ssGSEA, xCELL, TIMER and TIP, were used to calculate the abundances of TIICs in the tumor microenvironment. Based on Auslander’s study, we identified 22 immunosuppressive checkpoints with potential for therapeutic intervention (40). The T-cell inflammation score was calculated based on the mRNA expression of 18 genes. Immune and stromal scores were evaluated with the ESTIMATE R package (41).

### Identification and functional enrichment analysis of differentially expressed genes (DEGs)

The median expression level of TANK was used as the cutoff for dividing all patients into the high and low TANK expression groups. With the limma R package, TANK-related DEGs between the two groups (42) in the TCGA and CGGA-693 cohorts were identified. Adjusted *P* < 0.05 and |log (fold change) |>1 were considered the criteria for identifying DEGs. Gene set enrichment analysis (GSEA) was performed with GSEA software (vision 3.0) (http://www.broadinstitute.org/gsea) to explore the potential mechanism of TANK.

### Development and validation of a TANK-associated risk score by LASSO and machine learning

In the TCGA and CGGA-693 cohorts, univariate Cox regression analysis of the DEGs was performed using the survival R package. The TANK-associated prognostic model was established by using the LASSO procedure to identify 13 prognostic markers from among the 347 TANK-related DEGs significantly associated with prognosis by the R package “glmnet” in the TCGA cohort.

The root mean squared error (RMSE) was a measure of how well the machine learns the model, and was calculated by taking the square root of the average of the residuals (errors not explained by the regression equation) over the total sample size. The support vector machine (SVM) model selection of hyper-parameters was made based on lowest RMSE values (43). Using SVM regression, 10 genes were selected from 13 prognostic markers.

Individual risk scores were calculated based on the Cox regression coefficient (β) and mRNA expression levels. The training and validation sets were divided in the TCGA cohort at a ratio of 7:3. An R package was used to assess the statistical performance of the prognostic model. Additionally, the TANK-associated risk score was validated as a prognostic indicator and performer in the TCGA internal validation set, TCGA-all set, CGGA-693 set, CGGA-301 set, CGGA-325 set, GSE16011 dataset, and Rembrandt dataset.

### Statistical analysis

Pearson or Spearman correlation analysis was performed to investigate correlations between variables. The *t* test was used to compare continuous variables fitting a normal distribution between binary groups. Kaplan-Meier curves were generated for prognostic analyses based on categorical variables, and the log-rank test was used to estimate statistical significance. *P* < 0.05 was used as the criterion for significance, and all tests were two-sided.

## Results

### The expression pattern of TANK

**Figure 1:**
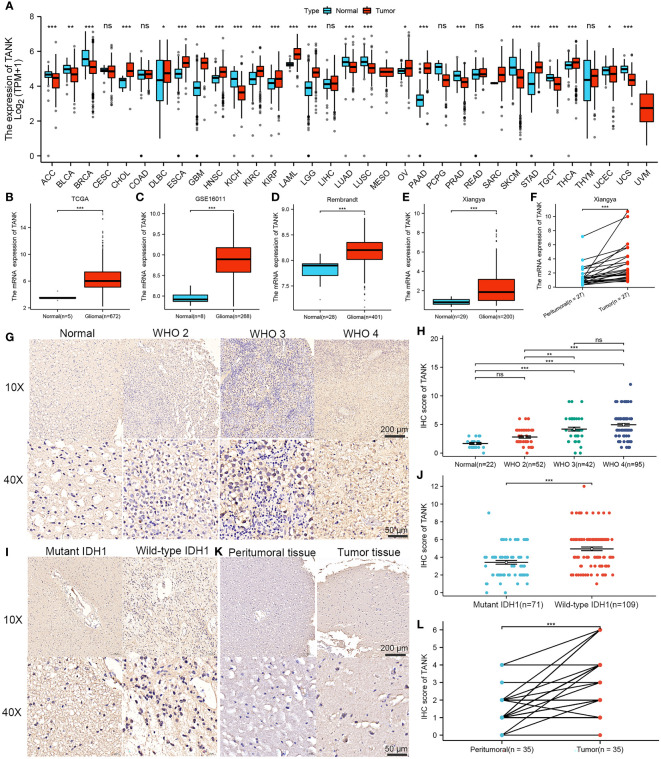
Elevated expression of TANK in glioma. **(A)** Differential expression of TANK between tumor and normal tissues from the TCGA dataset; **(B-E)** The expression level of TANK in the normal tissues and glioma tissues in the TCGA cohort **(B)**, GSE16011 **(C)**, Rembrandt cohort **(D)** and Xiangya cohort **(E)**; **(F)** The expression of TANK in glioma and peritumor tissues was analyzed by RT-qPCR; **(G-J)** The expression level of TANK in gliomas with different WHO grades **(G, H)**, and wild-type and mutant IDH1 **(I, J)** was analyzed by immunohistochemical staining; **(K, L)** The expression of TANK in glioma and peritumor tissues was analyzed by IHC (-no significance, *P < 0.05, **P < 0.01, and ***P < 0.001).

By integrating GTEx data with TCGA data, we were able to increase the number of normal tissue samples. TANK levels in various tumor tissues, including LGG and GBM tissues, were markedly higher than those in nontumor tissues (*P* < 0.05, **Figure 1A**). Analysis of the TCGA cohort showed higher TANK expression in tumor tissues than in normal brain tissues, and analyses of the GSE16011 and Rembrandt cohorts validated this observation (*P* < 0.001, **Figures 1B–D**). Additionally, we examined the expression pattern of TANK in four cohorts of patients with glioma. In the TCGA cohort, higher grade glioma tissues expressed significantly higher levels of TANK than lower grade glioma tissues (*P* < 0.001, **Figure S1A**). Results similar to those observed in the CGGA-693, Rembrandt and GSE16011 datasets were also observed (*P* < 0.05, **Figures S1B–D**). TANK expression was higher in gliomas with wild-type IDH than in those with mutant IDH in the three cohorts (*P* < 0.05, **Figures S1E–G**). TANK was generally highly expressed in GBM (*P* < 0.05, **Figures S1H–K**). We examined TANK expression in 29 normal tissues and 200 fresh tumor tissues, as well as in 27 paired tumor samples and peritumor tissues by qPCR. In the in-house cohort, TANK expression was high in glioma tissues (*P* < 0.001, **Figure 1E**). Furthermore, the tumor tissues exhibited significantly higher levels of TANK expression than the matched peritumor tissues (*P* < 0.001, **Figure 1F**). High expression of TANK was found at a significantly higher rate in WHO grade IV gliomas, wild-type IDH1 gliomas, and GBM (*P* < 0.001, **Table 2**), consistent with the above results. Immunohistochemical analysis of the tissue microarrays revealed that TANK was upregulated in gliomas (*P* < 0.05, **Figures 1G, H**). TANK was significantly enriched in glioma with WHO grade IV, wild-type IDH1, and GBM (*P* < 0.05, **Figures 1G–J**) (*P* < 0.05, **Table 2**). Furthermore, TANK was significantly overexpressed in tumor tissues in 35 tumor-peritumor tissue pairs (*P* < 0.001, **Figures 1K, L**). Thus, higher TANK expression is associated with more malignant glioma phenotypes.

**Table 2:** Association of TANK expression with clinical parameters in two cohorts.

Characteristic	Cohort1 (n=200)		*P*	Cohort2 (n=203)		*P*
	Low (n=100)	High (n=100)		Low (n=128)	High (n=75)	
Age, Mean ± SD	42.95 ± 15.20	45.02 ± 16.34	0.212	43.91 ± 15.19	46.91 ± 16.98	0.107
Gender, n (%)			0.789			0.062
Female	43 (21.5%)	40 (20%)		48 (23.6%)	39 (19.2%)	
Male	54 (27%)	58 (29%)		80 (39.4%)	36 (17.7%)	
Unknown	3 (1.5%)	2 (1%)		0 (0%)	0 (0%)	
WHO grade, n (%)			0.010			< 0.001
2	37 (18.5%)	20 (10%)		48 (23.6%)	4 (2%)	
3	20 (10%)	16 (8%)		28 (13.8%)	14 (6.9%)	
4	31 (15.5%)	53 (26.5%)		42 (20.7%)	53 (26.1%)	
Unknown	12 (6%)	11 (5.5%)		10 (4.9%)	4 (2%)	
IDH1 status, n (%)			0.005			< 0.001
Mutant	42 (21%)	25 (12.5%)		57 (28.1%)	14 (6.9%)	
Unknown	14 (7%)	8 (4%)		20 (9.9%)	3 (1.5%)	
Wild-type	44 (22%)	67 (33.5%)		51 (25.1%)	58 (28.6%)	
Histology, n (%)			0.018			< 0.001
Astrocytoma	45 (22.5%)	29 (14.5%)		66 (32.5%)	19 (9.4%)	
Gangliocytoma	3 (1.5%)	2 (1%)		4 (2%)	1 (0.5%)	
GBM	29 (14.5%)	53 (26.5%)		43 (21.2%)	53 (26.1%)	
Oligodendroglioma	14 (7%)	10 (5%)		15 (7.4%)	2 (1%)	
Unknown	9 (4.5%)	6 (3%)				
Radiotherapy, n (%)			0.205			0.093
No	21 (10.5%)	29 (14.5%)		41 (20.2%)	14 (6.9%)	
Unknown	32 (16%)	22 (11%)		16 (7.9%)	14 (6.9%)	
Yes	47 (23.5%)	49 (24.5%)		71 (35%)	47 (23.2%)	
Chemotherapy, n (%)			0.243			0.438
No	19 (9.5%)	25 (12.5%)		37 (18.2%)	18 (8.9%)	
Unknown	32 (16%)	22 (11%)		16 (7.9%)	14 (6.9%)	
Yes	49 (24.5%)	53 (26.5%)		75 (36.9%)	43 (21.2%)	
Laterality, n (%)			0.295			0.238
Both	3 (1.5%)	2 (1%)		2 (1%)	4 (2%)	
Center	38 (19%)	48 (24%)		60 (29.6%)	29 (14.3%)	
Middle	6 (3%)	4 (2%)		3 (1.5%)	4 (2%)	
Right	50 (25%)	46 (23%)		63 (31%)	38 (18.7%)	
Unknown	3 (1.5%)	0 (0%)		0 (0%)	0 (0%)	
Tumor location, n (%)			0.440			0.903
Brainstem	2 (1%)	1 (0.5%)		2 (1%)	1 (0.5%)	
Cerebellar	6 (3%)	4 (2%)		6 (3%)	1 (0.5%)	
Frontal	46 (23%)	41 (20.5%)		56 (27.6%)	37 (18.2%)	
Insular	2 (1%)	4 (2%)		3 (1.5%)	2 (1%)	
Occipital	3 (1.5%)	8 (4%)		9 (4.4%)	5 (2.5%)	
Parietal	8 (4%)	10 (5%)		12 (5.9%)	10 (4.9%)	
Sellar	2 (1%)	1 (0.5%)		1 (0.5%)	1 (0.5%)	
Temporal	25 (12.5%)	30 (15%)		37 (18.2%)	17 (8.4%)	
Thalamus	3 (1.5%)	1 (0.5%)		2 (1%)	1 (0.5%)	
Unknown	3 (1.5%)	0 (0%)		0 (0%)	0 (0%)	

### TANK is an indicator of poor prognosis in glioma

**Figure 2:**
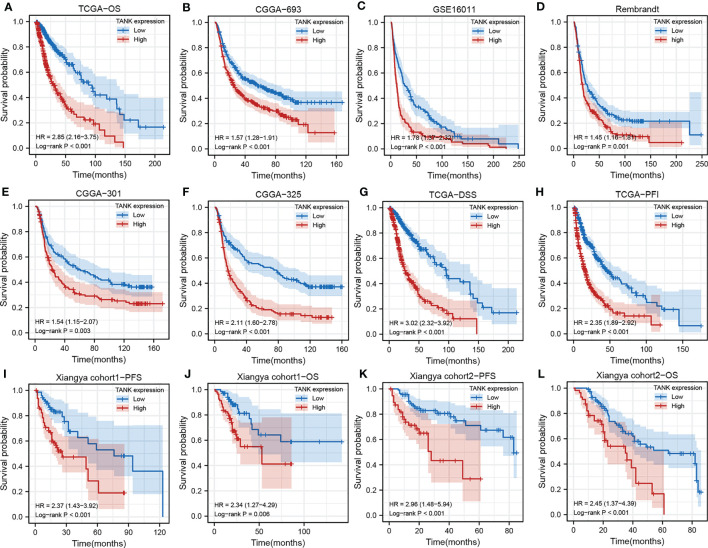
TANK is an unfavorable prognostic marker in glioma. **(A-F)** Kaplan-Meier curves displaying the correlations between TANK expression and OS in glioma patients in TCGA **(A)**, CGGA-693 **(B)**, GSE16011 **(C)**, Rembrandt **(D)**, CGGA-301 **(E)** and CGGA-325 **(F)** datasets; **(G, H)** Kaplan-Meier curves showing the correlations between TANK expression and DSS **(G)** and PFI **(H)** in the TCGA cohort; **(I, J)** Kaplan-Meier curves showing the correlations between TANK expression and PFS **(I)** and OS **(J)** in in-house cohort 1 based on qPCR data; **(K, L)** Kaplan-Meier curves showing the correlations between TANK expression and PFS **(K)** and OS **(L)** in in-house cohort 2 based on immunohistochemical data; *P* values were calculated by the log-rank test, and *P* < 0.05 was considered significant.

Survival curves were used to explore the prognostic implications of TANK expression in glioma in the six cohorts, and the results consistently revealed that TANK is an indicator of poor prognosis in glioma (TCGA, HR=2.85 (2.16-3.75); CGGA-693, HR=1.57 (1.28-1.91); GSE16011, HR=1.78 (1.37-2.32); Rembrandt, HR=1.45 (1.16-1.8); CGGA-301, HR=1.54 (1.15-2.07); CGGA-325, HR=2.11 (1.60-2.78), log-rank test *P* < 0.05, **Figures 2A–F**). When further exploring the relationship of TANK expression with DSS and PFS, patients with high expression of TANK were found to have shorter survival times (DSS, HR=3.02 (2.32-3.92); PFS, HR=2.35 (1.89-2.92), log-rank test *P* < 0.05, **Figures 2G, H**). In our in-house cohort of 158 glioma patients, we found that glioma patients with high TANK expression generally had shorter OS and PFS times than patients with low TANK expression by qPCR (PFS, HR=2.37 (143-3.92); OS, HR=2.34 (1.27-4.29); log-rank test *P* < 0.05, **Figures 2I, J**). Similarly, significant prognostic differences were observed in other in-house cohorts using IHC, and the survival time for patients with glioma with high TANK expression was shorter than that of patients with low TANK expression (PFS, HR=2.96 (1.48-5.94); OS, HR=2.45 (1.37-4.39); log-rank test *P* < 0.05, **Figures 2K, L**). Thus, TANK is a marker of unfavorable prognosis in glioma.

### The correlations of TANK with immunological parameters

**Figure 3:**
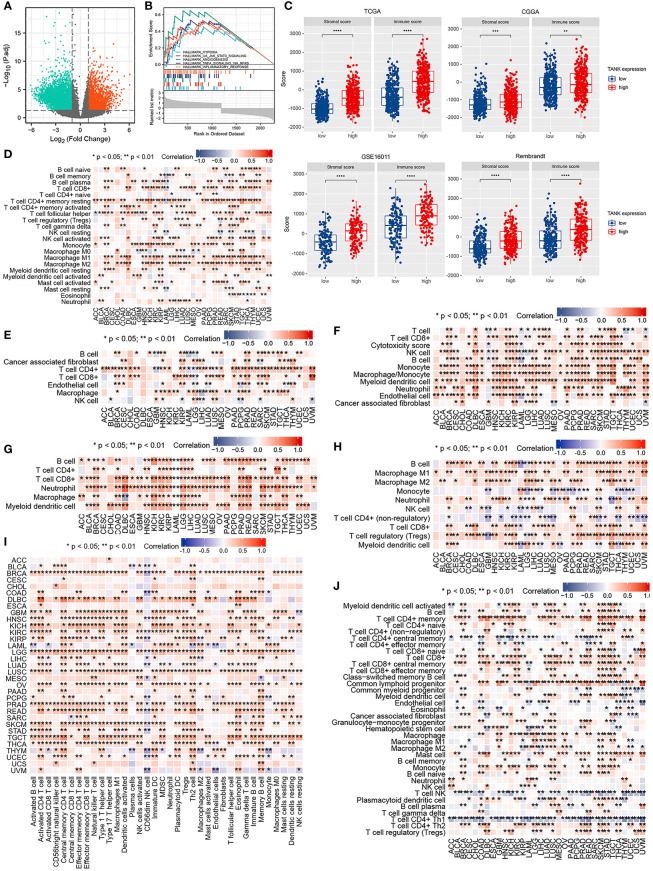
Immune relevance of TANK. **(A)** The volcano plot shows differentially expressed genes between the low- and high- TANK groups in the TCGA cohort; **(B)** GSEA of gliomas with low and high expression of TANK in the TCGA cohort; thresholds of a nominal *P* < 0.05 and an FDR < 25% were used to determine the significance of the enrichment score (ES); **(C)** The associations between the stromal and immune scores and TANK expression in the TCGA cohort, CGGA-693 cohort, GSE16011, and Rembrandt cohort; differences between the two groups were compared by Student’s t test, and the *P* values are labeled above each boxplot with asterisks (**P < 0.01, ***P < 0.001, and ****P < 0.0001); **(D-J)** Correlation of TANK expression with immune cell infiltration, as evaluated using seven algorithms (TIMER, EPIC, xCELL, CIBERSORT, QUANTISEQ, MCP-counter, and ssGSEA).

Considering that TANK expression is correlated with glioma malignancy, we inferred that abnormal expression of TANK might promote the progression of glioma. Among the DEGs, 2902 were significantly upregulated and 1370 were significantly downregulated in the TCGA cohort (**Figure 3A**). TANK’s underlying pathways were further clarified using GSEA. Gliomas with high TANK levels exhibited enrichment in immunomodulatory pathways, including “hypoxia”, “angiogenesis”, “inflammatory response”, “NF-kappaB signaling”, and “IL6/STAT3 signaling” in the TCGA cohort (**Figure 3B**). Next, the complex microenvironment of glioma was assessed by using the ESTIMATE algorithm (41). Furthermore, we found that gliomas with high levels of TANK consistently exhibited higher immune and stromal scores than those with low levels of TANK in four cohorts (*P* < 0.05, **Figure 3C**), indicating that TANK may regulate immune and stromal cells. We assessed cell infiltration in 33 cancers using seven algorithms. TANK expression was negatively correlated with infiltration of antitumor immune cells such as CD8^+^ T cells, follicular helper T cells and NK cells (*P* < 0.05, **Figures 3D–J**). In summary, TANK plays a vital role in the tumor microenvironment.

### TANK shapes an immunosuppressive microenvironment in glioma

**Figure 4:**
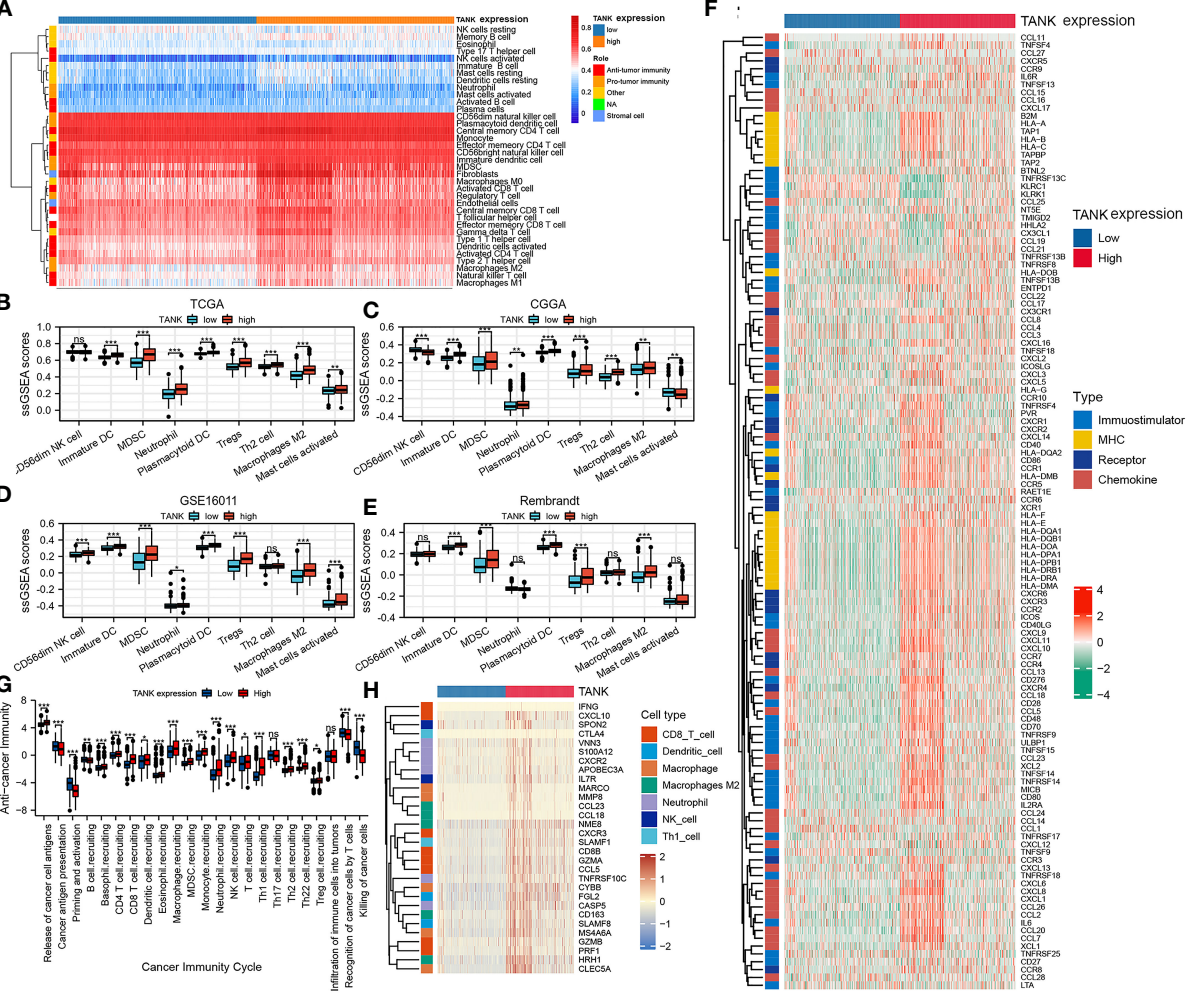
TANK shapes an immunosuppressive TME in glioma. **(A)** The landscape of immune cells and stromal cells in the low- and high- TANK groups in the TCGA cohort; **(B–E)** The association between TANK expression and the abundances of seven types of protumor immune cells in the TCGA cohort **(B)**, CGGA-693 cohort **(C)**, GSE16011 **(D)**, and Rembrandt cohort **(E)**; **(F)** Differences in the expression of 122 immunomodulators (chemokines, receptors, MHC, and immunostimulators) between the high- and low-TANK groups in glioma; **(G)** Differences in the various steps of the cancer immunity cycle between the high- and low-TANK groups; **(H)** Differences in the markers of immune cells between the high- and low-TANK groups in the TCGA cohort (- no significance, *P < 0.05, **P < 0.01, and ***P < 0.001).

An increasing number of studies have shown that glioma is a brain tumor characterized by an immunosuppressive microenvironment formed by immunosuppressive cells, which limits the prognosis of tumor therapy (44, 45). Given that TANK may remodel the tumor microenvironment through immunobiological processes, the distribution of 35 immune cell types in gliomas with high and low expression of TANK was examined in the TCGA cohort (**Figure 4A**). Correlation analysis between TANK expression and infiltration of protumor immune cells in the TCGA cohort revealed that gliomas with high TANK expression contained more immunosuppressive cells, except for CD56dim NK cells (*P* < 0.05, **Figure 4B**). Other cohorts showed similar results (**Figures 4C-E**, *P* < 0.05). However, there was no difference in the abundance of Th2 cells in gliomas in other cohorts (**Figures 4C-E**, *P* > 0.05). Additionally, though the difference in neutrophil infiltration was not observed in the Rembrandt cohort (**Figure 4E**, *P* > 0.05), gliomas with high-expression TANK had higher neutrophilic infiltration than those with low-expression TANK in other cohorts (**Figures 4B-D**, *P* < 0.05).

In the high-TANK group, most MHC molecules were overexpressed, indicating an enhanced ability to present and process antigens. In addition, the levels of CXCL9, CXCL10, and CCR3, which increase the recruitment of CD8+ T cells into the microenvironment of glioma, were increased in gliomas with high TANK expression (46, 47). Chemokines and paired receptors, including CCL2 and CCR2, were upregulated in TANK-expressing gliomas (**Figure 4F**). The recruitment of effector TIICs is promoted by these chemokines and receptors. Due to the complex and diverse functions of the chemokine system, studies on the relationship between TANK and individual chemokines are insufficient to elucidate the overall immune effect of TANK in the microenvironment.

The cancer immunity cycle includes seven steps: release of cancer cell antigens (Step 1), cancer antigen presentation (Step 2), priming and activation (Step 3), trafficking of immune cells to tumors (Step 4), infiltration of immune cells into tumors (Step 5), recognition of cancer cells by T cells (Step 6), and killing of cancer cells (Step 7). The activity of the tumor immune cycle is a direct result of the function of the chemokine system and immunomodulators (48). In the high-TANK group, the activities of most steps were downregulated, including Step 1, Step 3, and Step 4 (macrophage recruitment, Th1 cell recruitment, NK cell recruitment, and Th17 recruitment), was downregulated (**Figure 4G**). Consequently, these reduced activities may reduce the level of effector TIIC infiltration into the microenvironment. Interestingly, the activity of cancer cell recognition by T cells was downregulated in the low-TANK group. The activity of Step 7 (killing of cancer cells) was downregulated in the high-TANK group. Immune cell markers were upregulated in the high-TANK group compared with the low-TANK group (**Figure 4H**).

### TANK predicts clinical response and therapeutic opportunities

**Figure 5:**
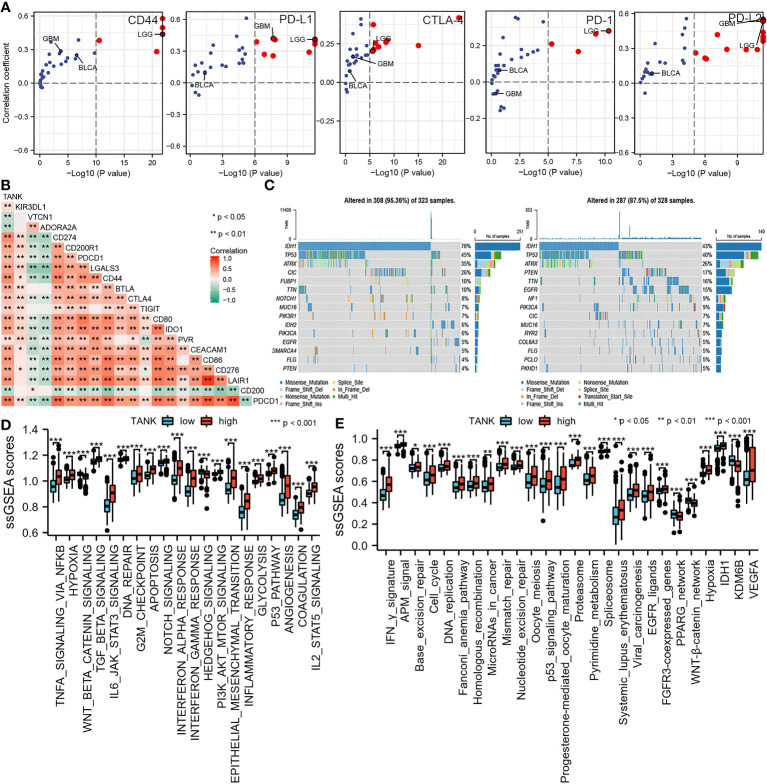
TANK predicts clinical response and therapeutic opportunities. **(A)** The associations between TANK expression and the mRNA expression of several immune checkpoints across pan-cancer; **(B)** The association between TANK expression and the mRNA expression of several immune checkpoints in glioma; **(C)** Mutation profiles in the low- and high-TANK groups in the TCGA cohort; **(D)** Correlations between TANK expression and the enrichment scores of several oncogenic pathways; **(E)** Differences in the enrichment scores of immunotherapy-related pathways between the high- and low-TANK groups in the TCGA cohort. *p<0.05, **p<0.01, and ***p<0.001.

Pan-cancer analyses showed that the immunological role of TANK is critical for determining the types of cancers that may benefit from anti-TANK immunotherapy. We found that expression of TANK was mutually exclusive with that of several immune checkpoints, including PD-L1, PD-1, CD44, CTLA-4, and PD-L2 (**Figure 5A**; **Table S1**). TANK expression was positively correlated with the ssGSEA scores of most immunotherapy-associated signatures (**Figure 5B**). In addition, genetic abnormalities are classical biomarkers of the anti-PD-1/PD-L1 therapeutic response (49). Mutations in the high-TANK group were shown using a waterfall plot (**Figure 5C**). IDH1 and ATRX were not frequently mutated in gliomas with high TANK expression (IDH1, 43% and ATRX, 26%) compared with those with low TANK expression (IDH1, 78% and ATRX, 35%), while PTEN, TTN and EGFR were more frequently mutated in gliomas with high TANK expression (PTEN, 17%, TTN, 16% and EGFR, 15%) than in those with low-level TANK (PTEN, 4%, TTN, 10% and EGFR, 5%). Several oncogenic pathways cooperatively form the immunosuppressive microenvironment of glioma. Therefore, blocking these pathways suppresses the formation of an immunosuppressive microenvironment. We found that immunosuppressive oncogenic pathways were significantly enriched in gliomas with high expression of TANK (*P* < 0.05, **Figures 5D, E**).

### TANK expression is positively correlated with CD163, CD11b, PD-1, PD-L1, CD40, STAT3 and HIF1A expression in glioma

**Figure 6:**
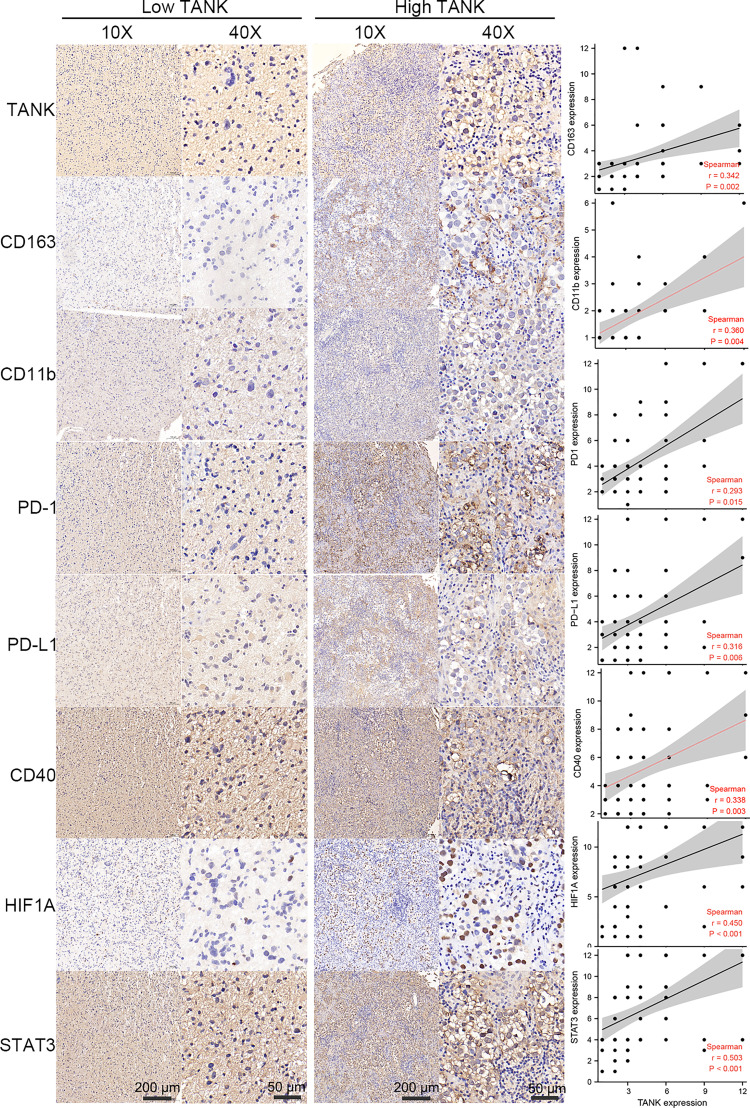
TANK expression is positively correlated with CD163, CD11b, PD-1, PD-L1, CD40, STAT3 and HIF1A expression in glioma. As surface markers of M2 macrophages and neutrophils, IHC shows that CD163 and CD11b are positively correlated with the expression of TANK (CD163, Spearman r = 0.342, *P* < 0.05; CD11b, Spearman r = 0.360, *P* < 0.05). As important immunosuppressive molecules, PD-1, PD-L1 and CD40 expression increased with the increase of TANK expression (PD-1, Spearman r = 0.293, *P* < 0.05; PD-L1, Spearman r = 0.316, *P* < 0.05; CD40, Spearman r = 0.338, *P* < 0.05). TANK was found to be positively related to HIF1A expression (Spearman r = 0.450, *P* < 0.05), a core molecule of the hypoxia-induced signaling pathway, and STAT3 (Spearman r = 0.503, *P* < 0.05), a key molecule of IL6/STAT3 signaling pathway.

As mentioned above, the expression of TANK was correlated with the abundances of immune cells, including neutrophils and macrophages, and the expression of immune checkpoints. TANK was also found to be involved in a variety of signaling pathways, including “hypoxia” and “IL6/STAT3 signaling” (**Figure 3B**). Therefore, we further analyzed the correlation between TANK expression and the expression of CD163 and CD11b, surface markers of M2 macrophages and neutrophils, respectively by IHC. M2 macrophages and neutrophils are important components of the glioma microenvironment and have been reported to be closely related to the prognosis of patients (50). TANK expression was positively correlated with the expression of CD163 and CD11b (CD163, Spearman r = 0.342, *P* < 0.05; CD11b, Spearman r = 0.360, P < 0.05, **Figure 6**). Considering that PD-1/PD-L1 and CD40 are important immunosuppressive molecules, we investigated the relationship between TANK expression and the expression of these molecules at the protein level. IHC showed that PD-1, PD-L1 and CD40 expression increased with increasing TANK expression (PD-1, Spearman r = 0.293, *P* < 0.05; PD-L1, Spearman r = 0.316, *P* < 0.05; CD40, Spearman r = 0.338, *P* < 0.05, **Figure 6**). Finally, TANK expression was found to be positively related to the expression of HIF1A, a core molecule of the hypoxia-induced signaling pathway, and STAT3, a key molecule of the IL6/STAT3 signaling pathway (HIF1A, Spearman r = 0.450, *P* < 0.05; Spearman r = 0.503, *P* < 0.05, **Figure 6**). These observations confirm that TANK may be involved in regulating the complex tumor microenvironment.

### Development, validation, and evaluation of the TANK-associated risk score

**Figure 7:**
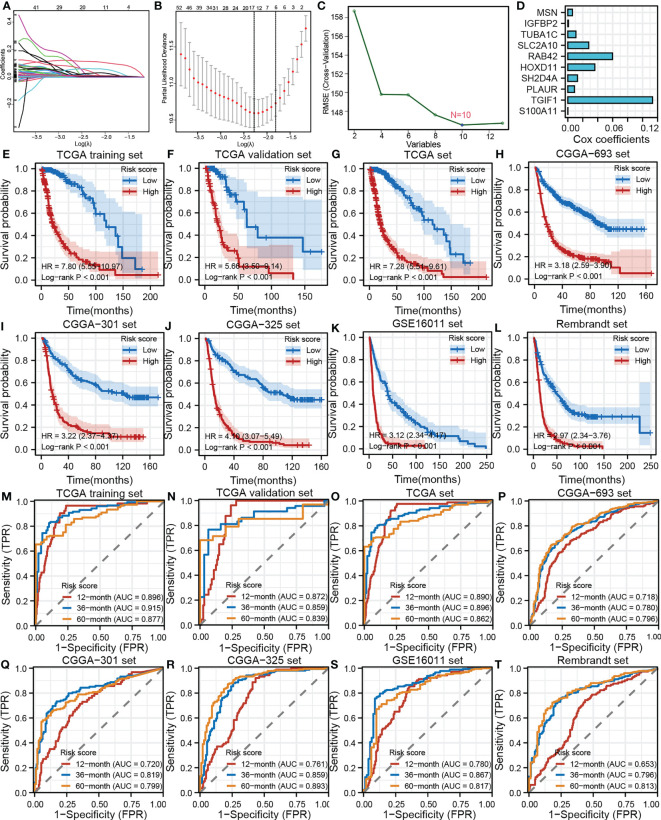
Development, validation and evaluation of the TANK-associated risk score. **(A)** The partial likelihood deviance distribution of the LASSO coefficient; **(B)** Partial likelihood deviance determined by the LASSO regression model; **(C)** Identification of hub genes by SVM; **(D)** An ensemble of 10 TANK-associated signatures with the Cox regression coefficients; **(E-L)** Kaplan-Meier curves show the correlation between the risk scores and overall survival of patients in the TCGA training set **(E)**, TCGA internal validation set **(F)**, whole TCGA set **(G)**, CGGA-693 cohort **(H)**, CGGA-301 **(I)**, CGGA-325 **(J)**, GSE16011 **(K)**, and Rembrandt cohorts **(L)**; *P* values were calculated by the log-rank test, and *P* < 0.05 was defined as the cutoff criterion; **(M-T)** Time-dependent ROC analysis of survival at 12 months, 36 months, and 60 months showed the predictive accuracy of the TANK-associated prognostic model in the TCGA training set **(M)**, TCGA internal validation set **(N)**, whole TCGA set **(O)**, CGGA-693 cohort **(P)**, CGGA-301 **(Q)**, CGGA-325 **(R)**, GSE16011 **(S)**, and Rembrandt cohorts **(T)**. AUC, area under the curve; ROC, receiver operating characteristic.

In this study, we identified 892 overlapping TANK-associated DEGs in the CGGA-693 and TCGA cohorts (**Table S2**). Among these DEGs, we also identified 299 TANK-associated DEGs significantly associated with prognosis (**Table S3**). Then, LASSO Cox regression model and SVM were applied to select the 10 most useful factors for developing a prognostic model in the TCGA training set (**Figures 7A–C**) and obtain a TANK-associated risk score for each patient based on the mRNA expression of thirteen genes and the corresponding LASSO Cox coefficients (**Figure 7D**). Patients with low-risk scores had significantly longer overall survival times than those with high-risk scores in the TCGA training set, TCGA internal validation set and TCGA set (TCGA training set, HR = 7.80 (5.55-10.97), *P* < 0.001; TCGA validation set, HR = 5.66 (3.50-9.14), *P* < 0.001; TCGA set, HR = 7.28 (5.51-9.61), *P* < 0.001, **Figures 7E–G**). The CGGA-693 cohort, CGGA-301, CGGA-325, GSE16011, and Rembrandt cohorts were used as external validation sets. The results revealed that the risk score could effectively divide patients into two distinct groups in these external validation cohorts. Patients in the high-risk group had a significantly poorer prognosis than those in the low-risk group (CGGA-693, HR = 3.18 (2.59-3.90), *P* < 0.001; CGGA-301, HR = 3.22 (2.37-4.37), *P* < 0.001; CGGA-325, HR = 4.10 (3.07-5.49), *P* < 0.001; GSE16011, HR = 3.12 (2.34-4.17), *P* < 0.001; Rembrandt, HR = 2.97 (2.34-3.76), *P* < 0.001, **Figures 7H–L**). The predictive accuracy of the risk score was well validated in the TCGA training set, TCGA internal validation set and TCGA set. The AUC of the risk score was more than 0.80 for survival at 12, 36, and 60 months in the TCGA training set, TCGA internal validation set and TCGA set (**Figures 7M–O**). Similarly, the AUC of the risk score was more than 0.80 for survival at 12, 36, and 60 months in the CGGA-693 cohort, CGGA-301, CGGA-325, GSE16011 and Rembrandt cohort (**Figures 7P–T**).

## Discussion

In this study, TANK was highly expressed in glioma (*P* < 0.05, **Figure 1**), as confirmed by qPCR and IHC (*P* < 0.05, **Figure 1**). In addition, the qPCR and IHC results indicated that higher TANK expression was associated with more malignant phenotypes in glioma (*P* < 0.05, **Figure 1**, **Table 2**). Furthermore, TANK was identified as a marker of poor prognosis in glioma. In two in-house cohorts, glioma patients with high expression of TANK generally had shorter OS and PFS times than those with low expression of TANK, as determined by qPCR and IHC (log-rank test *P* < 0.05, **Figures 2I–L**). Previous studies have shown that downregulation of TANK can arrest cells in S-phase and prevent tumor cell migration (36). Our results are consistent with previous results, indicating that TANK could play a protumorigenic role in glioma, and consistent with previous results. However, the clinical significance and expression pattern of TANK in glioma have not been reported. The possibility that TANK is a potential immunotherapeutic target for glioma needs further exploration.

TANK has been identified as a TRAF-interacting protein and can activate the NF-κB signaling pathway (26). The NF-κB pathway is indispensable for immune responses and inflammatory processes, as well as in activating survival and proinflammatory genes within the tumor microenvironment (51). Therefore, we hypothesized that TANK might be involved in the remodeling of the tumor microenvironment. GSEA showed that TANK was involved in immunoregulatory pathways in the TCGA cohort (**Figure 3B**). TANK was found to be closely and positively associated with the expression of HIF1A and STAT3 (HIF1A, Spearman r = 0.450, *P* < 0.05; Spearman r = 0.503, *P* < 0.05, **Figure 6**), a result that validated the above findings. In four cohorts, the immune and stromal scores were higher in gliomas with high TANK expression than in gliomas with low TANK expression (*P* < 0.05, **Figure 3C**). Pan-cancer immune infiltration analysis based on seven algorithms showed that TANK expression was closely correlated with infiltration of immunosuppressive cells (*P* < 0.05, **Figures 3D–J**). Importantly, the abundances of most immunosuppressive cells were higher in gliomas with high TANK expression in the four cohorts (*P* < 0.05, **Figures 4B–E**). TANK was negatively related to various immunomodulators (**Figure 4F**). In the high-TANK group, the activities of most of the steps were downregulated (**Figure 4G**). Subsequently, inactivity of these steps may weaken the infiltration of immune cells into the microenvironment. Therefore, we inferred that TANK shapes an inflamed TME in glioma. Although the role of TANK in regulating the tumor microenvironment of glioma has not been reported in previous studies, its important role in other tumors has been reported. Moreover, TANK expression was positively correlated with PD-L1, PD-1, and CD44 expression in various cancers (**Figure 5A**). The immunohistochemical results showed that PD-1, PD-L1 and CD40 expression increased with the increasing TANK expression in glioma (**Figure 6**).

Inhibiting oncogenic pathways blocks the formation of an immunosuppressive microenvironment, thereby reactivating cancer immunity. Most pathways were observably upregulated in the high-TANK group (*P* < 0.05, **Figures 5D, E**). Our observations provide insight for subsequent research on the mechanism by which TANK expression regulates immunity and lay a foundation for developing new treatment options. Similarly, for gliomas with high expression of TANK, one of the previous treatment methods was to transform the immunosuppressive microenvironment into an immune-activated state, thus triggering the anticancer immune response. The expression of inhibitory immune checkpoints may be upregulated by negative feedback regulation. Therefore, subsequent ICB therapy may reactivate suppressed anticancer immunity. This approach may enhance the efficacy of anti-TANK therapy and help trigger anticancer immunity. The combination of different ICB drugs with anti-TANK therapy is more effective than single therapy. Current therapeutic targets of ICB therapy, such as PD-L1and PD-1, are associated with each other in other tumors. Therefore, the combination of these drugs seems to have a synergistic effect. In contrast, TANK expression was significantly positively correlated with that of some ICB targets, suggesting complementary effects of anti-TANK and ICB therapy.

Finally, we established a TANK-related risk model to predict prognosis based on the expression of TANK-related genes. The TANK-associated risk score could effectively divide patients into two distinct groups, and patients in the high-risk group had a significantly worse prognosis than those in the low-risk group (log-rank test *P* < 0.001, **Figures 7E–L**). The risk score showed good predictive accuracy (**Figures 7M–T**).

However, there are limitations to our study. First, some contradictory findings need to be confirmed by carrying out reliable experiments. Second, the role of TANK in the tumor microenvironment and its underlying regulatory mechanisms need to be further explored. Third, more functional experiments are needed to validate the role of TANK in the glioma microenvironment, especially the immune microenvironment.

In conclusion, high expression of TANK indicates a malignant phenotype of glioma, predicts a poor prognosis and shapes an immunosuppressive tumor microenvironment. Combined anti-tank cancer immunotherapy may be a more effective strategy than monotherapy.

## Data availability statement

The datasets presented in this study can be found in online repositories. The names of the repository/repositories and accession number(s) can be found within the article/**Supplementary Materials**.

## Ethics statement

Informed consent was obtained from all patients, and this study was approved by the ethics committee of Xiangya Hospital, Central South University.

## Author contributions

HL, QZ, SL, and YG conceived and designed the study and analyzed the results. Other authors performed analysis procedures. QZ, SL, and YG, wrote the manuscript. HL provided materials and funding support. All authors contributed to the editing of the manuscript. All authors have read and agreed to the published version of the manuscript.

## Supplementary material

Supplementary Figure 1 Expression pattern of TANK in glioma. **(A–D)** The expression level of TANK in glioma with different WHO grades in the TCGA cohort **(A)**, CGGA-693 cohort **(B)**, GSE16011 **(C)**, and Rembrandt cohort **(D)**; **(E–G)** The expression level of TANK in glioma with wild-type and mutant IDH or IDH1 in the TCGA cohort **(E)**, CGGA-693 cohort **(F)**, and GSE16011 **(G)**; **(H–K)** The expression level of TANK in glioma with different histologies in the TCGA cohort **(H)**, CGGA-693 cohort **(I)**, GSE16011 **(J)**, and Rembrandt cohort **(K)**. *P* < 0.05 was considered significant. (- no significance, **P* < 0.05, ***P* < 0.01, and ****P* < 0.001).

Supplementary Table 1 The relationship between the expression of TANK and several immune checkpoints, including PD-L1, PD-1, CD44, CTLA-4, and PD-L2.

Supplementary Table 2 TANK-associated DEGs in the CGGA-693 and TCGA cohorts.

Supplementary Table 3 299 TANK-associated DEGs significantly associated with prognosis.
