# Correction: TANK shapes an immunosuppressive microenvironment and predicts prognosis and therapeutic response in glioma

**DOI:** 10.3389/fimmu.2026.1874735

**Published:** 2026-05-21

**Authors:** Shasha Li, Youwei Guo, Huijuan Hu, Na Gao, Xuejun Yan, Quanwei Zhou, Hui Liu

**Affiliations:** 1 National Engineering Research Center of Ophthalmology and Optometry, Eye Hospital, Wenzhou Medical University, Wenzhou, China; 2 Oujiang Laboratory (Zhejiang Lab for Regenerative Medicine, Vision and Brain Health), Wenzhou, China; 3 Department of Neurosurgery, Xiangya Hospital, Central South University, Changsha, China; 4 State Key Laboratory of Ophthalmology, Optometry and Visual Science, Eye Hospital, Wenzhou Medical University, Wenzhou, China; 5 Department of Geriatrics, National Key Clinical Specialty, Guangzhou First People’s Hospital, Guangzhou Medical University, Guangzhou, China; 6 The National Key Clinical Specialty, Department of Neurosurgery, Zhujiang Hospital, Southern Medical University, Guangzhou, China

**Keywords:** glioma, immunosuppressive microenvironment, immune infiltration, TANK, prognosis

There was a mistake in **Figure 2D** as published. During the preparation of the revised figure files, an earlier version of **Figure 2D** was inadvertently included due to a version mix-up. The corrected **Figure 2** and its caption appear below. The corrected panel was generated from the same dataset and analysis procedure used in the article, and this correction does not affect the statistical results, data interpretation, or scientific conclusions of the article.

**Figure 2:**
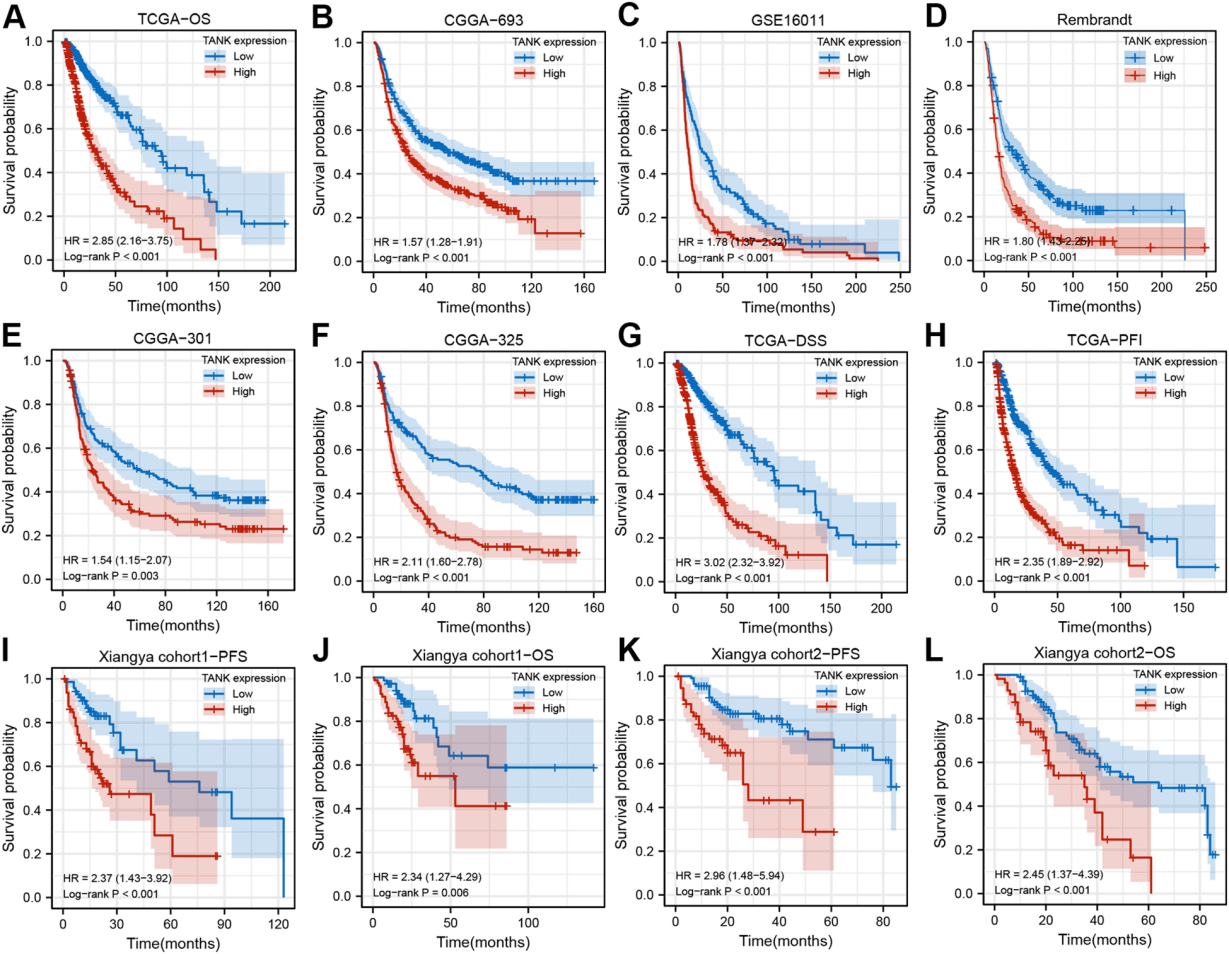


**Figure 2** TANK is an unfavorable prognostic marker in glioma. **(A–F)** Kaplan-Meier curves displaying the correlations between TANK expression and OS in glioma patients in TCGA **(A)**, CGGA-693 **(B)**, GSE16011 **(C)**, Rembrandt **(D)**, CGGA-301 **(E)** and CGGA-325 **(F)** datasets; **(G, H)** Kaplan-Meier curves showing the correlations between TANK expression and DSS **(G)** and PFI **(H)** in the TCGA cohort; **(I, J)** Kaplan-Meier curves showing the correlations between TANK expression and PFS **(I)** and OS **(J)** in in-house cohort 1 based on qPCR data; **(K, L)** Kaplan-Meier curves showing the correlations between TANK expression and PFS **(K)** and OS **(L)** in in-house cohort 2 based on immunohistochemical data; *P* values were calculated by the log-rank test, and *P* < 0.05 was considered significant.

Also, there was a mistake in **Figure 6**. During figure preparation, several representative immunohistochemistry images were incorrectly arranged, resulting in the incorrect placement of representative TANK, PD-1, and CD40 immunohistochemistry panels in the High TANK group. The corrected **Figure 6** and its caption appear below. This error affected only the representative immunohistochemistry images and did not affect the quantitative analyses, Spearman correlation coefficients, P values, or the scientific conclusions of the article.

**Figure 6:**
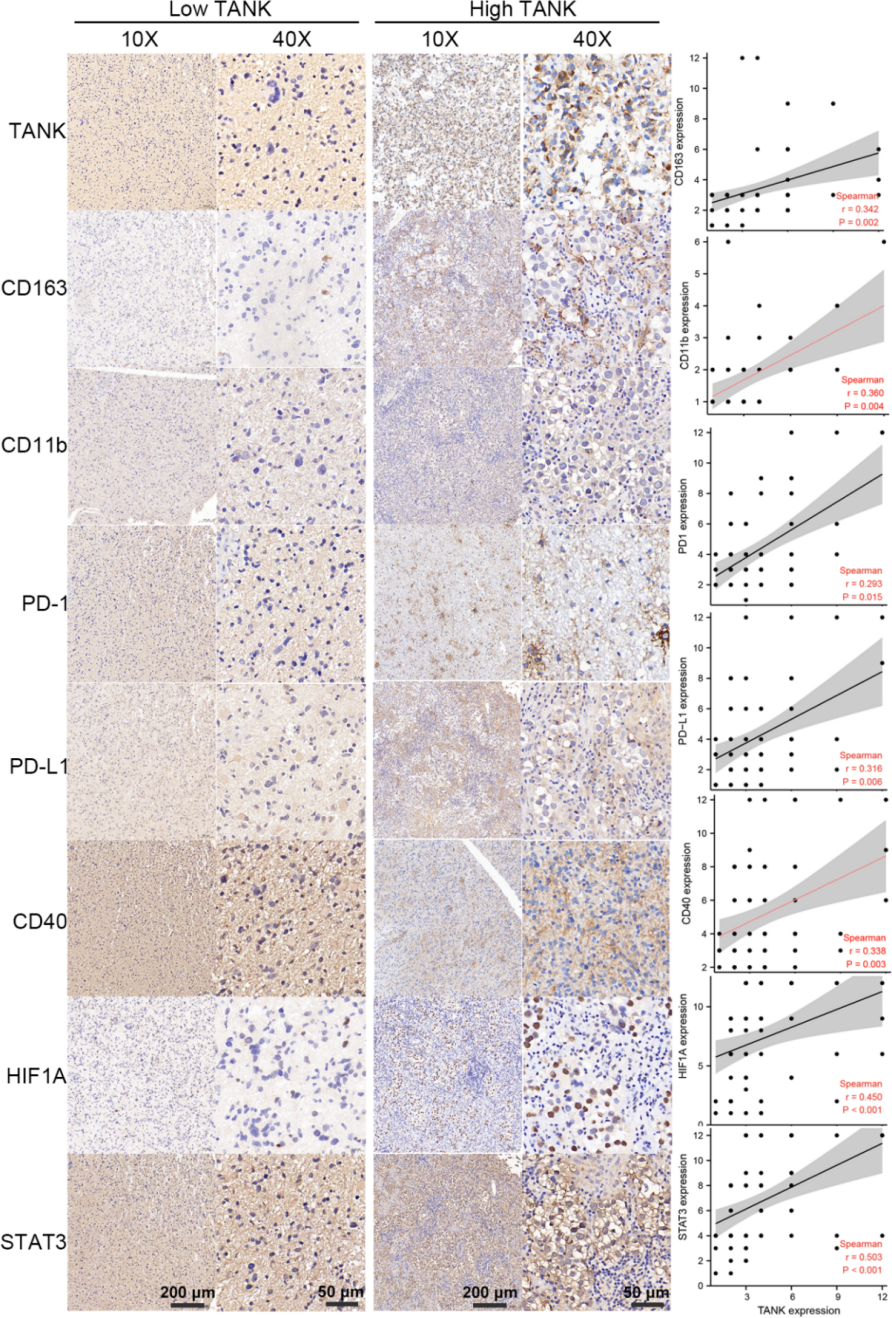


The original article has been updated.

**Figure 6** TANK expression is positively correlated with CD163, CD11b, PD-1, PD-L1, CD40, STAT3 and HIF1A expression in glioma. As surface markers of M2 macrophages and neutrophils, IHC shows that CD163 and CD11b are positively correlated with the expression of TANK (CD163, Spearman r = 0.342, *P* < 0.05; CD11b, Spearman r = 0.360, *P* < 0.05). As important immunosuppressive molecules, PD-1, PD-L1 and CD40 expression increased with the increase of TANK expression (PD-1, Spearman r = 0.293, *P* < 0.05; PD-L1, Spearman r = 0.316, *P* < 0.05; CD40, Spearman r = 0.338, *P* < 0.05). TANK expression was also positively correlated with HIF1A expression (Spearman r = 0.450, *P* < 0.05), a core molecule of the hypoxia-induced signaling pathway, and STAT3 (Spearman r = 0.503, *P* < 0.05), a key molecule of IL6/STAT3 signaling pathway.

