# Supplementary figures and images for: TANK shapes an immunosuppressive microenvironment and predicts prognosis and therapeutic response in glioma
Source: Front Immunol. 2023 May 5;14:1138203. doi: 10.3389/fimmu.2023.1138203 (PMC10196049; doi:10.3389/fimmu.2023.1138203)

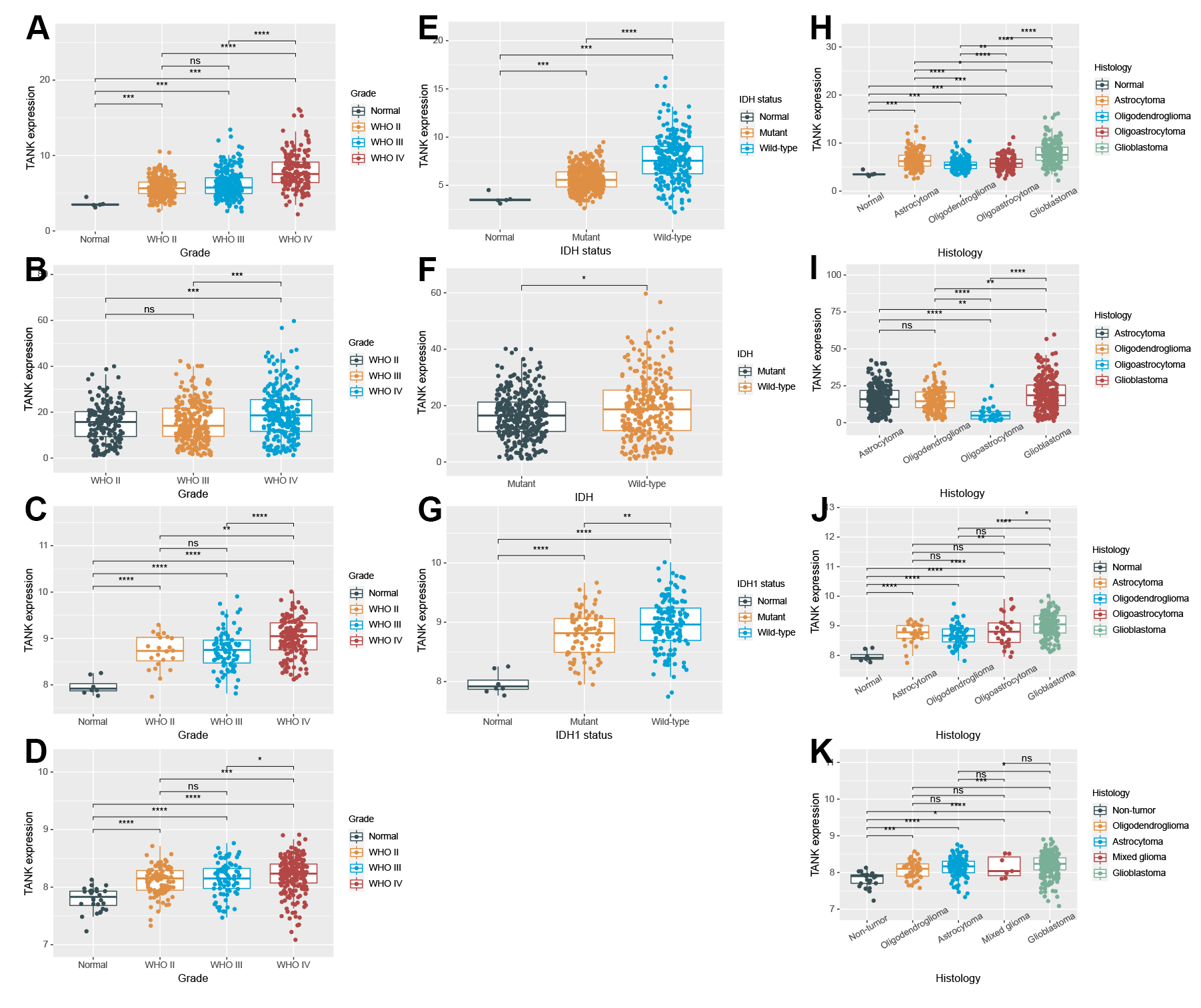

Supplement: Supplementary Figure 1 — Expression pattern of TANK in glioma. (A–D) The expression level of TANK in glioma with different WHO grades in the TCGA cohort (A), CGGA-693 cohort (B), GSE16011 (C), and Rembrandt cohort (D); (E–G) The expression level of TANK in glioma with wild-type and mutant IDH or IDH1 in the TCGA cohort (E), CGGA-693 cohort (F), and GSE16011 (G); (H–K) The expression level of TANK in glioma with different histologies in the TCGA cohort (H), CGGA-693 cohort (I), GSE16011 (J), and Rembrandt cohort (K). P < 0.05 was considered significant. (- no significance, *P < 0.05, **P < 0.01, and ***P < 0.001). [file Image_1.tif]
